# Supplementary material for: A thermostable type I-B CRISPR-Cas system for orthogonal and multiplexed genetic engineering
Source: Nat Commun. 2023 Oct 4;14:6193. doi: 10.1038/s41467-023-41973-5 (PMC10551041; doi:10.1038/s41467-023-41973-5)
Supplement: Supplementary file 1 — Supplementary Information [file 41467_2023_41973_MOESM1_ESM.pdf]

Supplementary material for:

A thermostable type I-B CRISPR-Cas system for orthogonal and multiplexed genetic engineering

Zhiheng Yang[1, 2, †], Zilong Li[2, †], Bixiao Li[2, †], Ruihong Bu[2, 3], Gao-Yi Tan[1], Zhengduo Wang[1], Hao Yan[2], Zhenguo Xin[2], Guojian Zhang[3], Ming Li[2, 4], Hua Xiang[2, 4], Lixin Zhang[1, *] and Weishan Wang[2, 4, *]

[1]State Key Laboratory of Bioreactor Engineering, and School of Biotechnology, East China University of Science and Technology (ECUST), Shanghai, 200237, China
[2]State Key Laboratory of Microbial Resources, Institute of Microbiology, Chinese Academy of Sciences, Beijing, 100101, China
[3]School of Medicine and Pharmacy, Ocean University of China, Qingdao 266003, China
[4]University of Chinese Academy of Sciences, Beijing 100049, China

[†] Contributed equally to this work.
* Corresponding author.

Email: wangws@im.ac.cn. Correspondence may also be addressed to Lixin Zhang, Email: lxzhang@ecust.edu.cn.

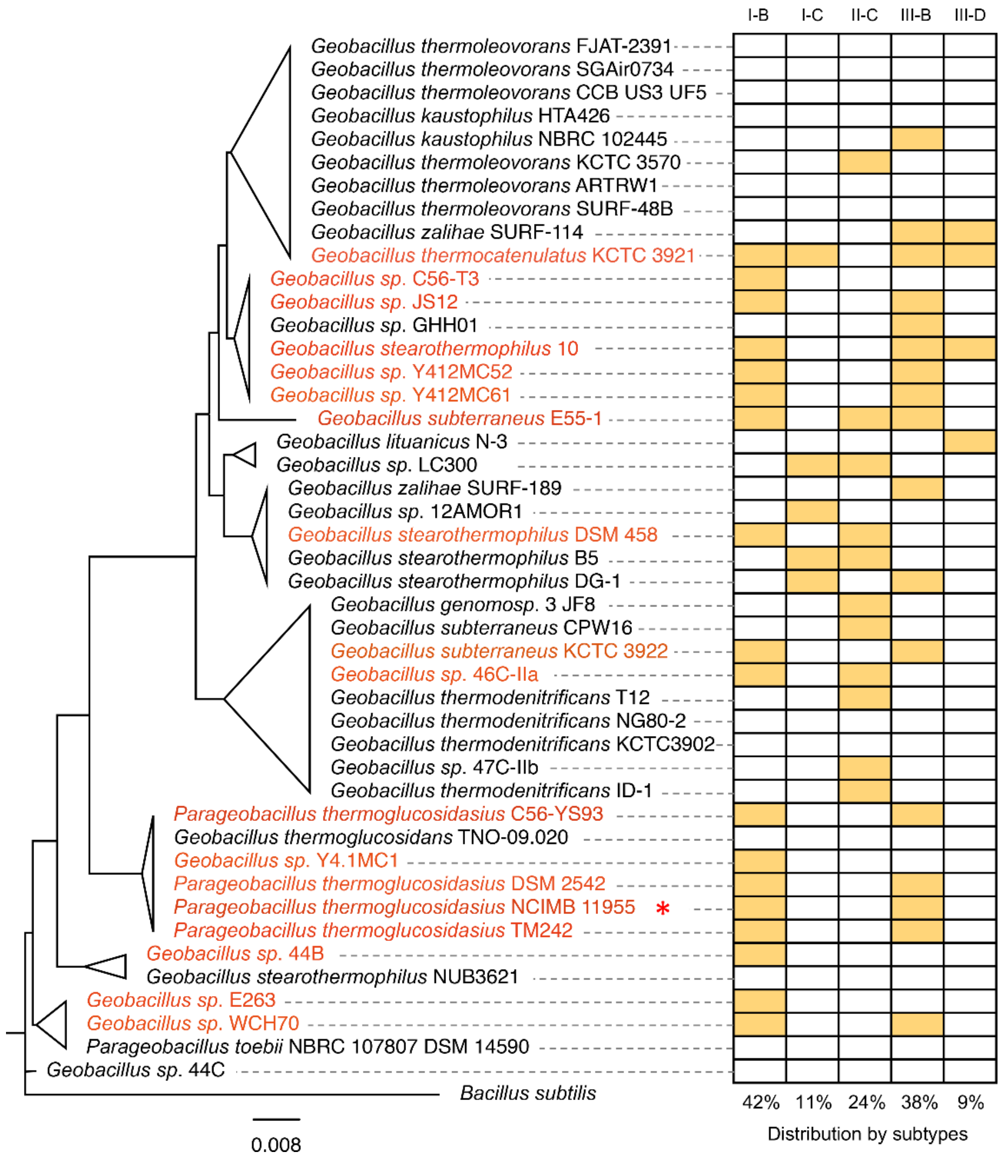

Supplementary Figure 1. Distribution of diverse CRISPR-Cas system with complete genome of thermophilic *Geobacillus* and *Parageobacillus*. Phylogenetic tree constructed by neighbor-joining algorithm from the distance matrix of 16S rDNA partial sequences of thermophilic *Geobacillus* and *Parageobacillus*. Class 1 CRISPR-Cas systems including type I-B, I-C, III-B, and III-C are dominant in this genus, whereas only type II-C belong to Class 2 systems CRISPR-Cas. Among them Type I-B is the most abundant, which accounts for 42% of total surveyed strains. The strains contain type I-B CRISPR-Cas system are indicate by uppercase red letters.

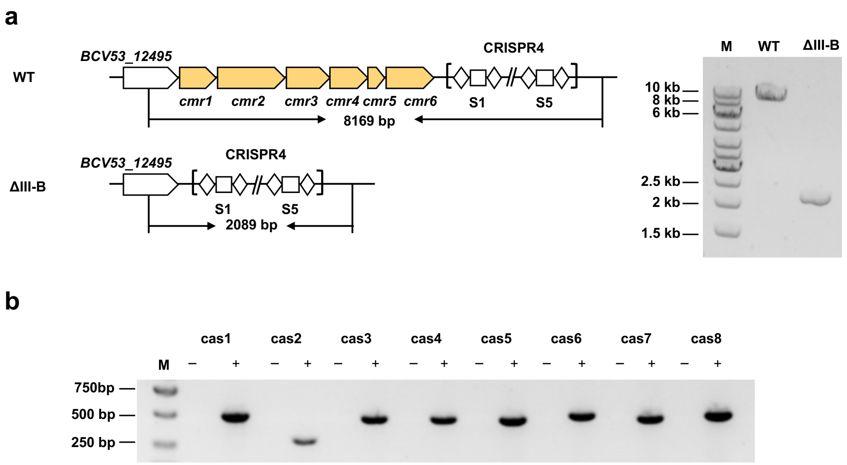

Supplementary Figure 2. Deletion of type III-B CRISPR-Cas system in *P. thermoglucosidasius*. (**a**) Left panel: Schematic of genetic information for wild-type (WT) and type III-B CRISPR-Cas system knockout strains (**Δ**III-B). Right panel: Confirming the deletion of all the type III-B Cas proteins from strain NCIMB 11955 by PCR. wild-type, 8169 bp; knockout mutant, 2089 bp. Lane M, DNA size marker. (**b**) Confirmation the expression of this type I-B CRISPR-Cas at transcriptional level in strain Y1 using RT-PCR. The symbol '–' indicates the sample before reverse transcription, and '+' indicates that after reverse transcription.

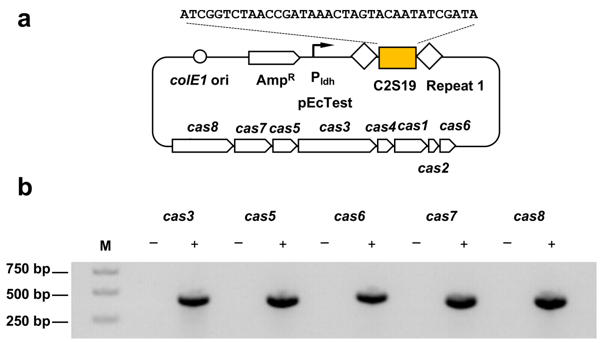

Supplementary Figure 3. Activity of the type I-B CRISPR-Cas in *E. coli* W3110. (**a**) Schematic of the plasmid used for transferring this type I-B CRISPR-Cas into *E. coli* W3110. The type I-B Cas effectors and the single-spacer mini-CRISPR were inserted into this plasmid. (**b**) Confirmation the expression of this type I-B CRISPR-Cas at transcriptional level in *E. coli*. Reverse transcription PCR were employed.

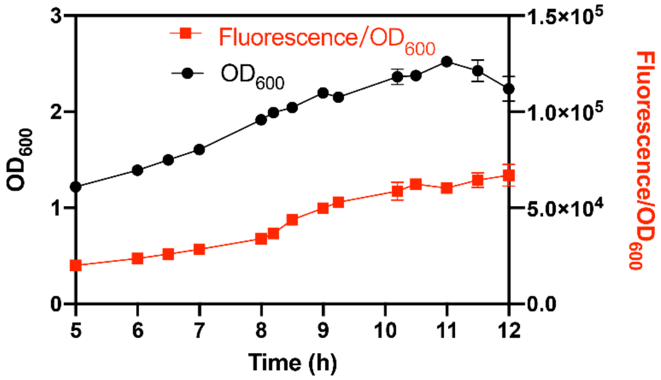

Supplementary Figure 4. Time-course expression of *sfgfp* driven by P_{ldh} promoter in strain Y2. The fluorescence intensity showed positive correlation with cell growth.

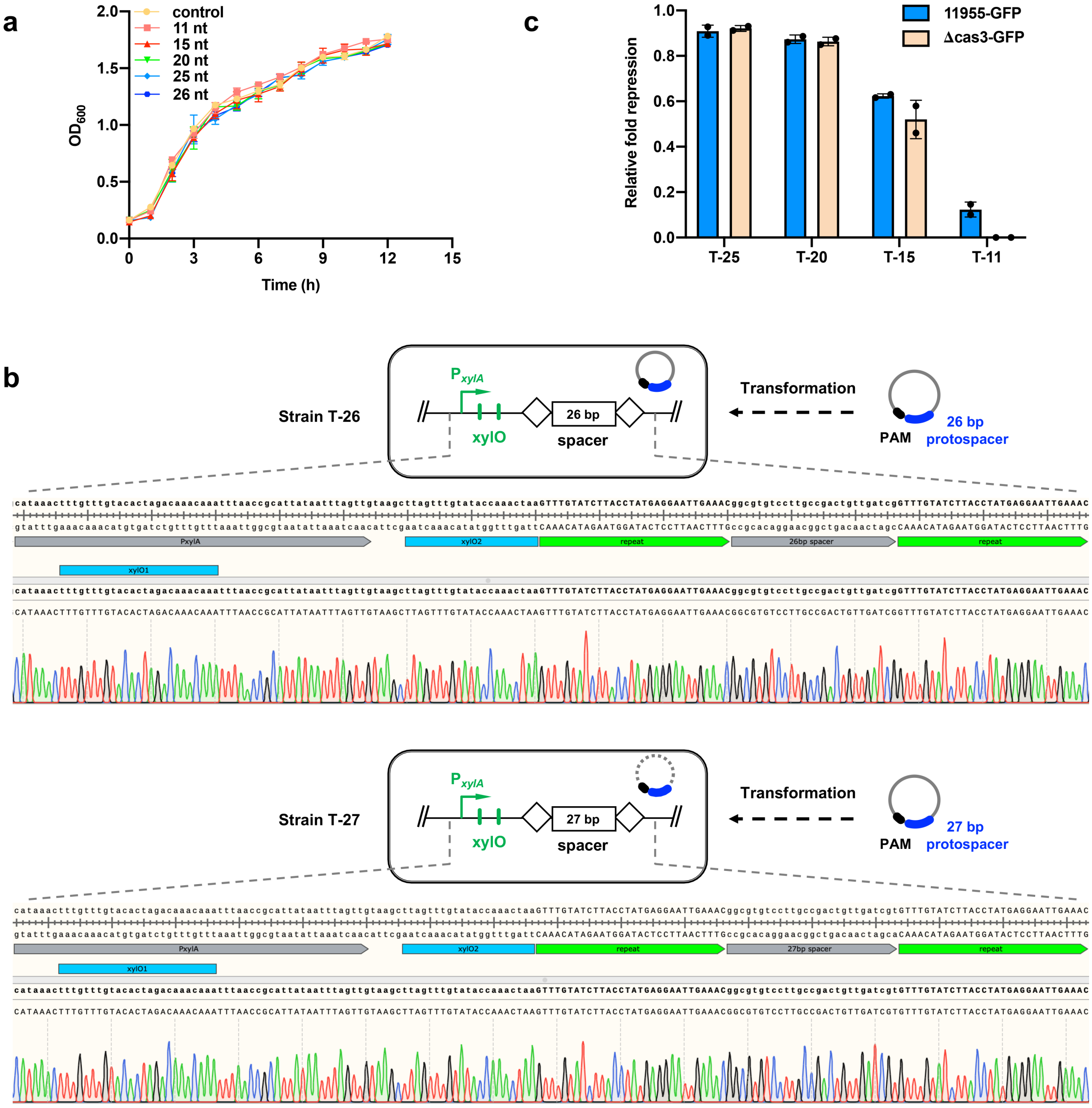

Supplementary Figure 5. Characterizing the interference and transcription repression of native type I-B system. (**a**) The growth curve of strain Y2 with 26-nt, 25-nt, 20-nt, 15-nt or 11-nt crRNA completely complementary to target *sfgfp* gene. The control strain containing pZH04 plasmid without crRNA. (**b**) The schematic of determine the switch point between interference and transcription repression. The rectangular shape signifies a derivative strain (T-26 or T-27) that has been knocked into an inducible mini CRISPR array. The array includes a truncated spacer, either 26-nt or 27-nt in length, which has been deliberately crafted to target the plasmids containing a protospacer of the corresponding length. The corresponding sequencing results of the mini CRISPR array are located underneath the rectangular shape. The green vertical line, repressor binding site xylO. The green arrow, A promoter that can be induced by xylose. The short black line, PAM sequence. The blue line, protospacer sequence. (**c**) The difference in transcription repression levels is compared between strains with and without Cas3 protein. 11955-GFP, the strain Y2 with Cas3 Protein in genome. **Δ**Cas3-GFP, the strain Y2 without Cas3 protein in genome. T-25/T-20/T-15/T-11, the strains contain a repressing plasmid expressing truncated spacers of different length. Data

are the mean of three biological repeats and are expressed as mean ± SD. Source data are provided as a Source Date file.

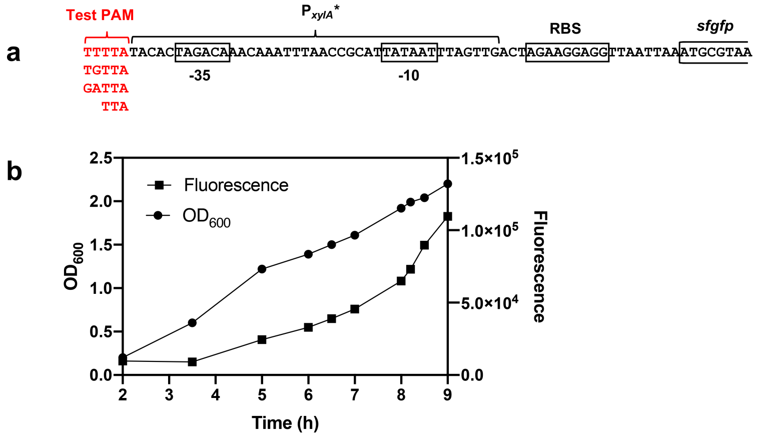

Supplementary Figure 6. Biosensor for screening the exact PAM. (**a**) sequence of the P_{xylA}* promoter. (**b**) Constitutive expression of sfGFP drived by P_{xylA}*. Source data are provided as a Source Date file.

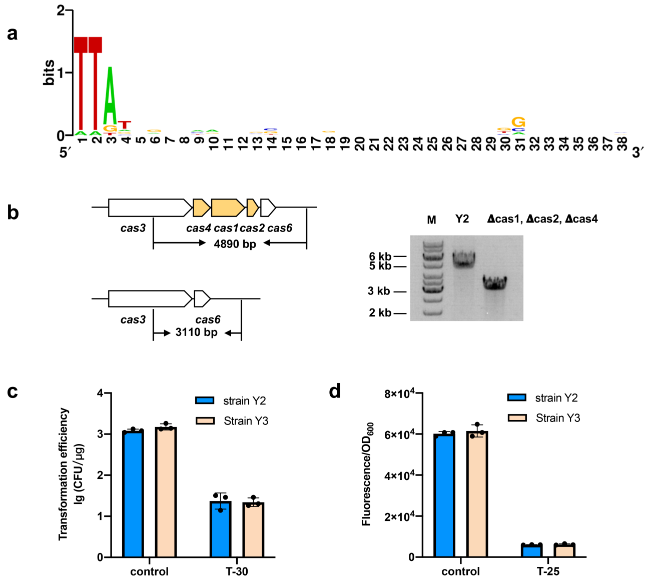

Supplementary Figure 7. Characterization of this type I-B CRISPR-Cas system. (**a**) Sequence logo showing the conserved PAM of TTA. The 38-nt upstream of each protospacer observed during truncated crRNA targeting *sfgfp* gene of Y1 genome were collected and analyzed with WebLogo (http://weblogo.berkeley.edu/logo.cgi). (**b**) Left panel: Schematic of genetic information for strain Y2 and knockout strain (**Δ**cas1, 2, 4). Right panel: Strain Y2, 4890 bp; knockout mutant, 3110 bp. Lane M, DNA size marker. (**c**) Testing the transformation efficiency of plasmid pT30 contain truncating to 30-bp's spacer to strain Y2 and Y3. Strain Y2 and Y3 containing control plasmid pZH04 as control strain. (**d**) Evaluating the ability of plasmid pT25 to transcription repression of sfGFP protein in the genomes of strain Y2 and Y3. Strain Y2 and Y3 containing control plasmid pZH04 as control strain. Data are the mean of three biological repeats and are expressed as mean ± SD. Source data are provided as a Source Date file.

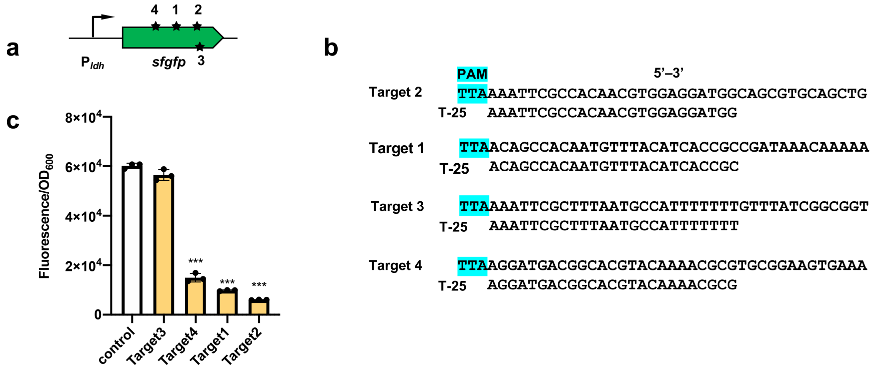

Supplementary Figure 8. Effect of crRNA location on transcriptional repression. (**a**) The position of truncated spacer targeting *sfgfp* gene. Spacer1, 2 and 4 targeting template strands of *sfgfp* gene. Spacer 3 targeting non-template strand of *sfgfp* gene. (**b**) Schematic of spacer1, 2, 3, and 4 with 25bp lengths contained plasmid pTT25-1, pTT25-2, pTT25-3 and pTT25-4, respectively. The blue background, the PAM sequence. Target1-4, the protospacer sequence. T-25, the spacer sequence. (**c**) Expression level of *sfgfp* gene in strain Y3 carrying plasmid pTT25-1, pTT25-2, pTT25-3 or pTT25-4 compared to that with the control plasmid pZH04. The exact p-values for Target3, Target4, Target1, and Target2 were 0.057, 2.53e−6, 1.26e−7, and 8.76e−8, respectively. Error bars indicate the standard deviation (SD) of three independent replicates. Statistical significance is calculated based on two-tailed Student's t-test (***$P < 0.001$). Source data are provided as a Source Date file.

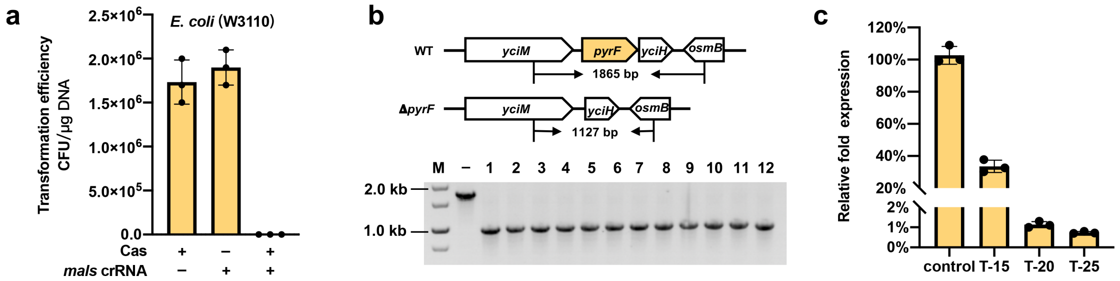

Supplementary Figure 9. Type I-B CRISPR-Cas system worked in mesophilic *E. coli*. (**a**) The transferred type I-B Cas system displayed a high DNA interference activity in strain W3110. (**b**) Confirmation of *pyrF* gene deletion by PCR. (**c**) Repression of *pyrF* gene characterized at transcription level using RT-qPCR. Data are the mean of three biological repeats and are expressed as mean ± SD. Source data are provided as a Source Date file.

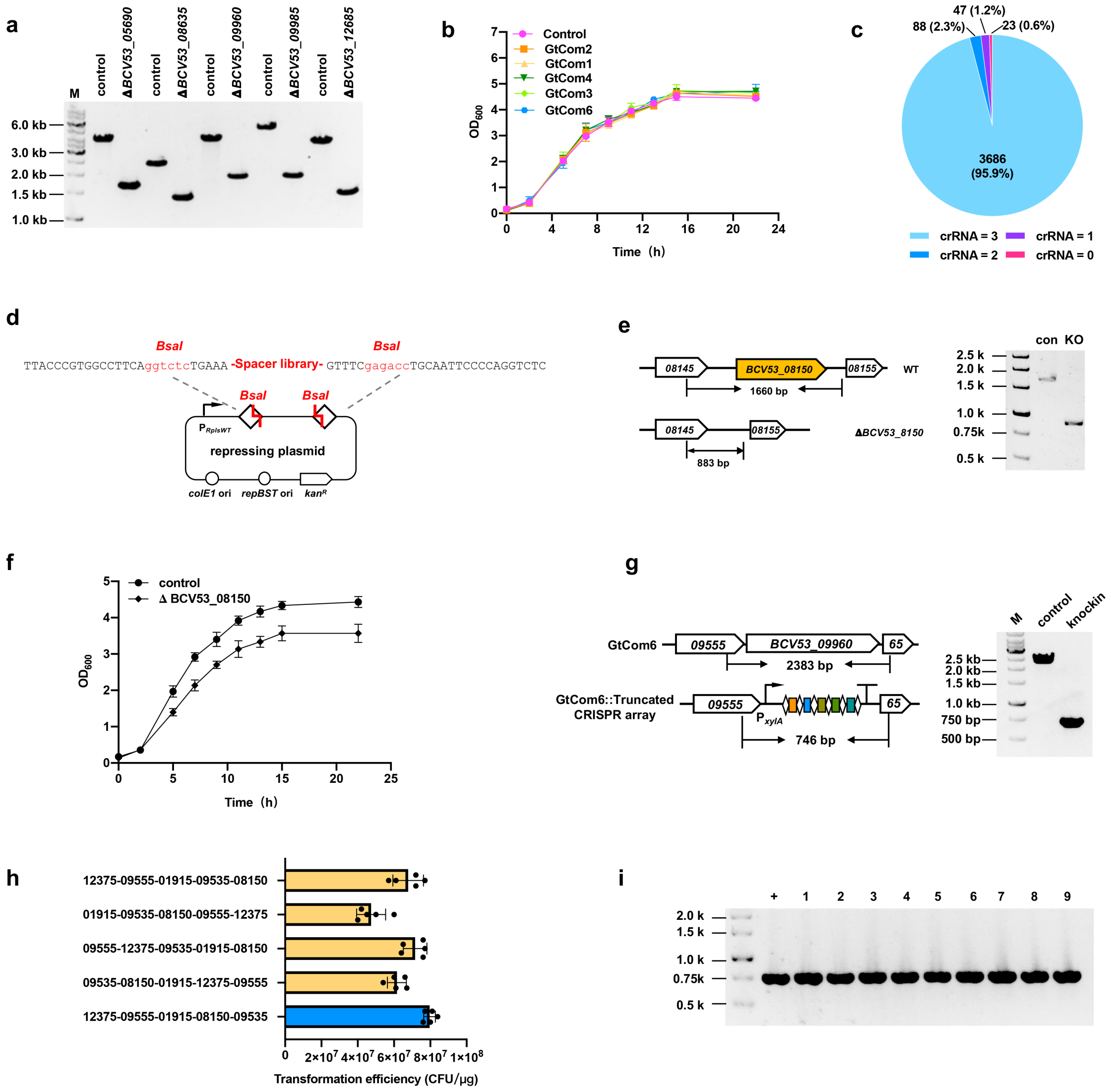

Supplementary Figure 10. Systematic improvement of transformation efficiency. (**a**) Confirmation of the deletion of *BCV53_05690, BCV53_08635, BCV53_09960, BCV53_09985* and *BCV53_12375*. (**b**) Growth curve of strains GtCom1–GtCom4 and GtCom6. Strain Y1 is used to control strain. (**c**) Statistics of genes with tgRNA numbers in our design. (**d**) Schematic of the repressing plasmid pZH04 used for construction of tgRNA library by golden gate assembly. The red bases indicate BsaI recognition site. (**e**) Left panel: Schematic of genetic information for control strain (Y1) and Knockout strain (KO). Right panel: Right panel: Confirming the deletion of *BCV53_08150* gene from strain Y1. Strain Y1, 1660 bp; knockout mutant, 883 bp. Lane M, DNA size marker. (**f**) Growth curve of strain Y1 and KO strain. (**g**) Left panel: Schematic of genetic information for strain GtCom6 and CRISPR array Knockin strain. Right panel: Confirming CRISPR array knock into strain GtCom6. Strain GtCom6, 2383 bp; knockin mutant, 746 bp. Lane M, DNA size marker. (**h**) Regarding the array order (12375-09555-01915-08150-09535), we arranged the tgRNAs based on their respective attributions to transformation efficiency (Fig. 5E), n = 5 biologically independent samples. We presume that the current order of the five tgRNAs represents the

optimal arrangement. To substantiate this presumption, we conducted tests using four alternative array orders in addition to the one mentioned, and the results indicated that the array order (12375-09555-01915-08150-09535) was the optimal arrangement among the five tested array orders. (**i**) Evaluating the stability of multiple tgRNAs on genome. Error bars indicate the standard deviation (SD) of three independent replicates. Source data are provided as a Source Date file.

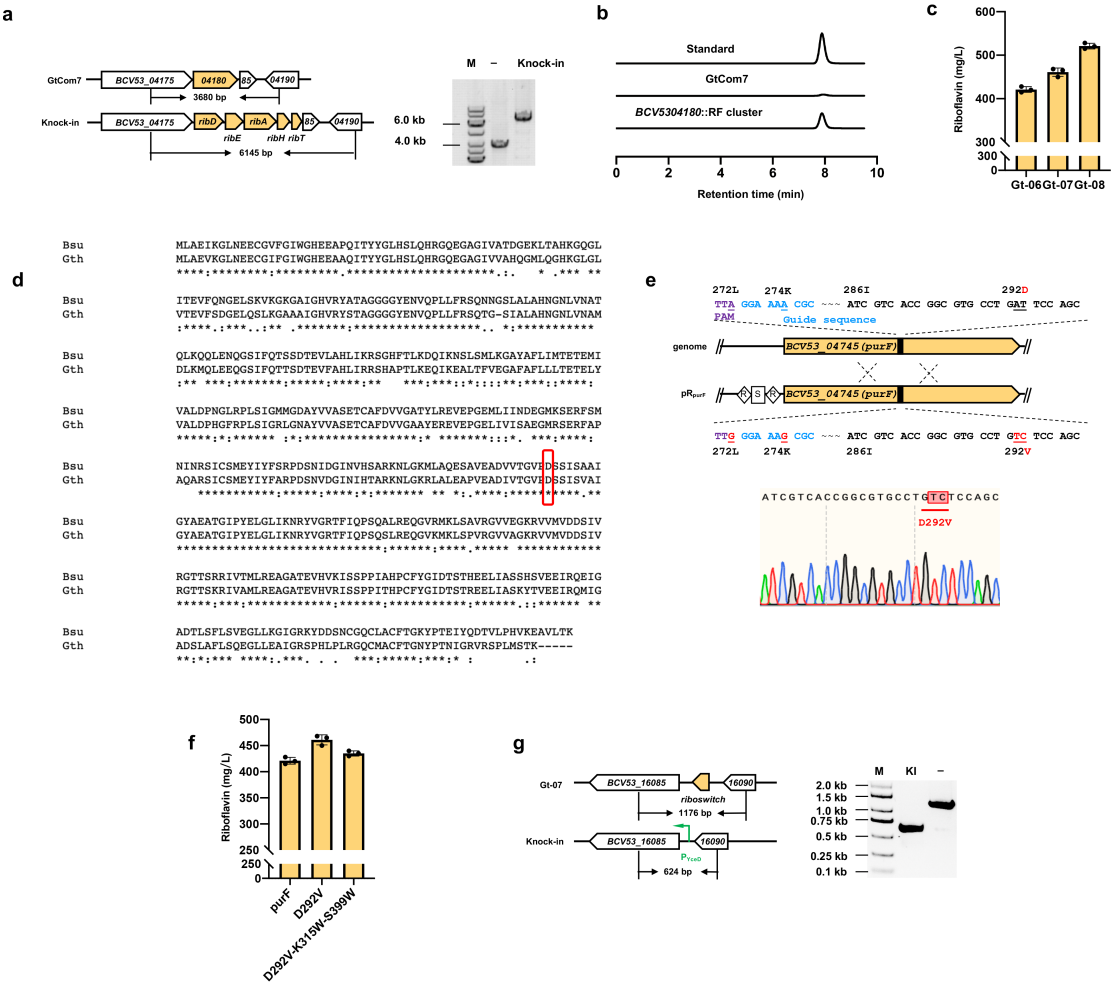

Supplementary Figure 11. Construction of riboflavin producer strain used for colorimetric screening. (**a**) Left panel: Schematic of genetic information for control strain (GtCom7) and knock-in strain (KI). Right panel: Confirmation of the knock-in of riboflavin biosynthetic gene cluster into strain GtCom7. Strain GtCom7, 3680 bp; knock-in mutant, 6145 bp. Lane M, DNA size marker. (**b**) Riboflavin production by knock-in riboflavin biosynthetic gene cluster into strain GtCom7 detected by HPLC. (**c**) Riboflavin titer of strain Gt-06 to Gt-08. (**d**) Alignment of PurF protein in *B. subtilis* and *P. thermoglucosidasius*, respectively. D292 in *P. thermoglucosidasius* equivalent to D293 in *B. subtilis*. (**e**) Confirmation of D292V point mutation of PurF by sequencing. (**f**) The riboflavin titer of different point mutation of PurF protein. (**g**) Left panel: Schematic of genetic information for control strain (Gt-07) and Knock-in strain (KI). Right panel: Confirmation of the desired P_{YceD} promoter knock-in mutant by PCR. Negative control, 1176 bp; knockout mutant, 624 bp. Error bars indicate the standard deviation (SD) of three independent replicates. Source data are provided as a Source Date file.

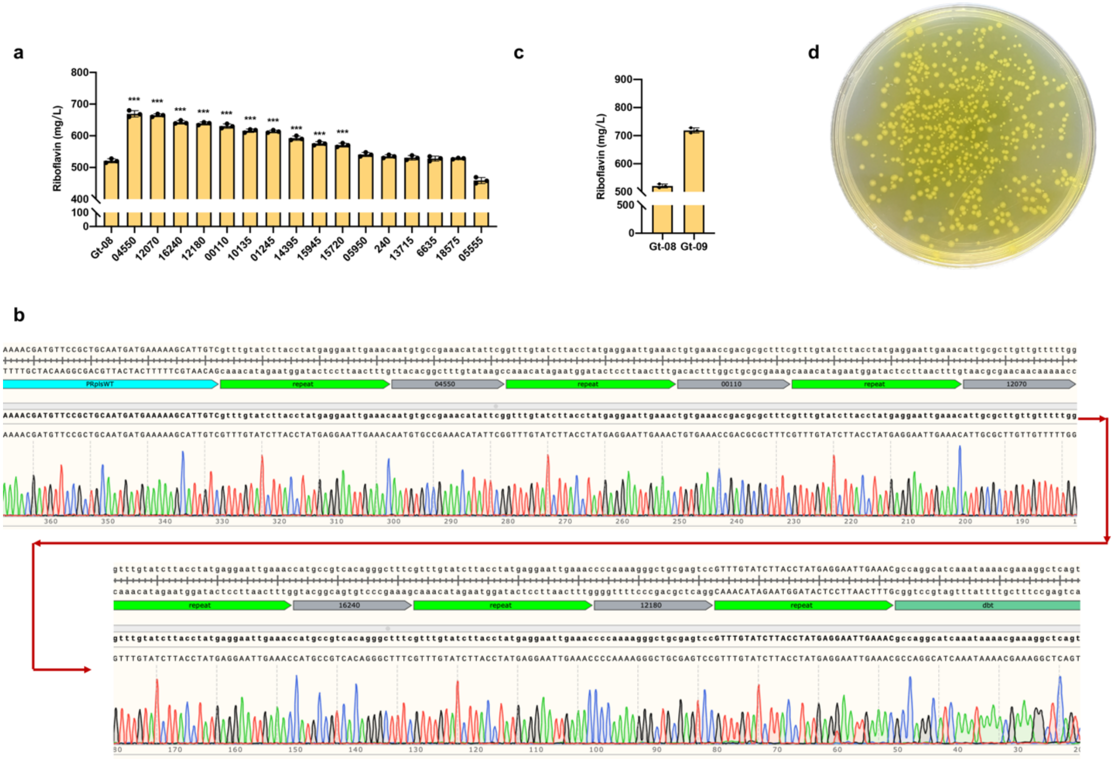

Supplementary Figure 12. Construction of thermophilic riboflavin cell factory by identification of and combination of multiple targets. (**a**) Colorimetric screen of riboflavin overproducing *P. thermoglucosidasius* strains. The exact *p*-values for *BCV53_04550*, *BCV53_12070, BCV53_16240, BCV53_12180, BCV53_00110, BCV53_10135, BCV53_01245, BCV53_14395, BCV53_15945, BCV53_15720,* and *BCV53_05950* were 2.83e−5, 6.23e−6, 1.61e−5, 1.39e−5, 3.86e−5, 4.05e−5, 3.13e−6, 0.00020, 0.00044, and 0.00064, respectively. (**b**) Confirming construction of CRISPR array with five truncated spacers by sequencing. (**c**) Riboflavin titer of strain Gt-08 and Gt-09. (**d**) Plate culture examination for identifying no bacterial contaminants. Error bars indicate the standard deviation (SD) of three independent replicates. Statistical significance is calculated based on two-tailed Student's *t*-test (****P* < 0.001). Source data are provided as a Source Date file.

Supplementary Table 1. Sequence of xylose-inducible promoter

Promoters	Sequence (5'-3')
P11585	gaataaatttttatcataaactttgtttgtacactagacaaacaaatttaaccgcattataatttagttgtaagttataaaaatttatggccaatatgaattttagttctatgacactattgtttttcgttgtatttatgatgaatgcttgtggacaggatgttgtaaactgaactgaaatggaagcgttaacaaaaaaggatgtacaggcaggacaagataaaaaataaagataggagctatgatcggcgcccttattatggccagcatcgataatgggatgagcatgatgaacatcgaaaccttttggcaatttattgtaaaaggattgattcttattatcgctgtctggattgatatatcgagtaaaagccgaactaactaattcatcggtgttttgaagattttagttaaaaatgctatttacattttgcgtaaatcccaatcattatgattggtttattctactaataaacatttattaataggaggaatttgcc
P_{xylA} (truncated P11585)	cataaactttgtttgtacactagacaaacaaatttaaccgcattataatttagttg

Supplementary Table 2. Statistics analysis of plasmid library via NGS

3844 protein-coding genes			Reads counts>0		Reads counts≥20	
library	Total read counts	Mapped read counts	sgRNA (ratio#)	Gene* (ratio#)	sgRNA (ratio#)	Gene^ (ratio#)
plasmid	3358558	2837421	99.96	99.89%	99.61%	98.72%

*Number of genes with at least one sgRNA that read count>0, ^Number of genes with at least one sgRNA that read count≥20, #Mapping ratio to the in silico library

Supplementary Table 3. Targets contributing to transformation efficiency

Name of Genes	Function
BCV53_12375	mannose-1-phosphate guanylyl transferase
BCV53_09555	DNA mismatch repair protein MutS
BCV53_01915	disulfide bond formation protein DsbA
BCV53_09535	spore coat protein
BCV53_08150	gluconate 5-dehydrogenase

Supplementary Table 4. Sequence of constitutive promoter P_{YceD}

Promoter	Sequence (5’-3’)
P_{YceD}	cgtttttgctctcccaagatttttttcagtgataaatgtcgatgaactgacaaccattatactgt aaagaaaaaaatattgacaaataggaaaacgaaacgtataattttctttgttgccttgaggt gatttttaaaaaaggaggcgagcgtcaaaaaaggaggcgagcgtc

Supplementary Table 5. Targets contributing to improve riboflavin production

Gene ID	Function	Titer % increase	Spacer (5'–3')
BCV53_04550	redox-sensing transcriptional repressor Rex	28.5	AATGTGCCGAAACATATTCG
BCV53_12070	preprotein translocase subunit SecA	27.6	ATTGCGCTTGTTGTTTTTGG
BCV53_16240	pyrroline-5-carboxylate reductase	23.3	ATTGCGCTTGTTGTTTTTGG
BCV53_12180	SAM-dependent methyltransferase	22.6	ATTGCGCTTGTTGTTTTTGG
BCV53_00110	3'-5' exonuclease KapD	21.0	TGTGAAACCGACGCGCTTTC
BCV53_10135	hypothetical protein	18.2	TTTGTTTAGACACATATACA
BCV53_01245	polysaccharide pyruvyl transferase	17.8	AGTGGTGAGAAACATGTCCG
BCV53_14395	glutathione ABC transporter permease GsiD	13.7	TTTGCAATTGCAATCATGGC
BCV53_15945	hypothetical protein	10.5	TCGCCGCCCGGCATAAGGCG
BCV53_15720	hypothetical protein	9.5	ATATGGAAGCCCGATCTTCC

Supplementary method 1. Riboflavin fermentation medium

To examine riboflavin production in 5-L bioreactor, engineered *P. thermoglucosidasius* was grown in riboflavin fermentation medium. The medium contains the following components: Na_2HPO_4 (6.78 g), KH_2PO_4 (3 g), K_2SO_4 (1.74g), Sodium citrate (0.516 g), $MgSO_4{\cdot}7H_2O$ (0.98 g), $Na_2MoO_4{\cdot}2H_2O$ (0.399 g), $CaCl_2$ (0.03 g), thiamin (0.01 g), yeast extract (20 g), Urea (3 g), Glucose (20 g), Glycine (2 g), Aspartic acid (1 g), biotin (3.1 mg) per liter of deionized water. Trace Metal Mix components: $ZnSO_4{\cdot}7H_2O$ (25 μM), $FeSO_4{\cdot}7H_2O$ (100 μM), $MnSO_4{\cdot}H_2O$ (50 μM), $CuSO_4{\cdot}5H_2O$ (5 μM), $CoSO_4{\cdot}7H_2O$ (10 μM), $NiSO_4{\cdot}6H_2O$ (16.85 μM), and H_3BO_3 (6.5 μM) in deionized water.

Supplementary method 2. Construction of plasmid used in this study

All protospacers and spacers utilized in this study were produced by annealing their corresponding primer pairs. For the plasmids of testing interference activity via targeting the identified protospacers, the protospacers C1S18, C1S18*, C2S19 and C3S32 including the 5 bp upstream sequence were inserted into the pUCG3.8 backbone digested with EcoRI and HindIII to generate plasmids pGeoth, pGeoth*, pVirus, and pPhage, respectively.

To deleting type III-B CRISPR-Cas system in *P. thermoglucosidasius* NCIMB 11955, the upstream and downstream fragments of the type III system were amplified from *P. thermoglucosidasius* genomic DNA using primer pair typeIII UF/typeIII UR and typeIII DF/typeIII DR, respectively. Then the two fragments and plasmid backbone treated with EcoRI and HindIII were assembled using Gibson Assembly to generate type III CRISPR-Cas system knockout plasmid pUB-type III KO.

For the test cleavage activity's plasmid pEcTest used in E. coli, I-B fragment was amplified from *P. thermoglucosidasius* genome using primer pair IB-F/IB-R. I-B fragment and plasmid backbone amplified from pThermocas9[1] using primer pair pNW33-F/pNW33-R were assembled to generate pNW33n-GTIB plasmid. Then, the pldh fragment amplified using primer pair pldh UF/pldh DR from pUB-sfGFP plasmid, mini-CRISPR fragment was amplified using annealed primer pair RSR UF/RSR UR. Finally, the two fragments and plasmid backbone amplified from pNW33n-GTIB plasmid using primer pair EcTest UF/EcTest DR were assembled using Gibson Assembly to generate plasmid pEcTest.

To generate the plasmid of *sfgfp* gene knock-in, the upstream and downstream fragments of the amylase gene were amplified from *P. thermoglucosidasius* genomic DNA using primer pair GFPKI UF/GFPKI UR and GFPKI DF/GFPKI DR, respectively. Next, the *ldh* promoter and *sfgfp* gene fragment was amplified using primer pair GFP-F/GFP-R from plasmid pUB-sfGFP. Finally, the three fragments and plasmid backbone treated with EcoRI and HindIII were assembled using Gibson Assembly to generate *sfgfp* gene knock-in plasmid pUB-GFPKI.

For the repressing plasmid pZH04, repeat 1 and repeat 2 fragments were obtained by annealed primer pair repeat 1F/repeat 1R and repeat 2F/repeat 2R, respectively. The promoter P_{RplsWT} was amplified from genomic DNA of *P. thermoglucosidasius* using primer pair pRplsWT-

F/pRplsWT-R. The terminator was amplified from plasmid pUB-sfGFP using primer pair T-F/T-R. Subsequently, the four fragments and plasmid backbone amplified from pUCG3.8 using primer pair pZH04V-F/pZH04V-R were assembled to generate plasmid pZH04.

For the plasmids that expressing a series of 38-nt crRNAs partially complementary to downstream of the TTTTA sequence of *sfgfp* target 2, the spacers P30, P25, P20, P15, and P11 were ligated into plasmid pZH04 digested with BsaI to obtain plasmid pP30-2, pP25-2, pP20-2, pP15-2 and pP11-2, respectively. Expressing a series of 38-nt crRNAs partially complementary to *sfgfp* target 1 and target 3 use similar approaches. For the plasmids that expressing a series of truncated crRNAs completely complementary to target 2, the spacers T30, T29, T28, T27, T26, T25, T20, T15, T11 were ligated into plasmid pZH04 digested with BsaI to obtain plasmid pT30, pT29, pT28, pT27, pT26, pT25, pT20, pT15 and pT11, respectively.

For editing plasmid pUB-T26KI and pUB-T27KI used to generate strain T-26 and strain T-27, Firstly, we constructed a platform plasmid pKIdonor. The upstream and a downstream fragments of donor DNA were amplified from *P. thermoglucosidasius* genomic DNA using primer pair donor UF/donor UR and donor DF/donor DR, respectively. Subsequently, the two fragments and plasmid backbone amplified from pUB-sfGFP using primer pair donor V-F/donor V-R were assembled to generate plasmid pdonor. Secondly, we obtain mini-CRISPR fragments though fusion PCR. P_{xylA} promoter containing another xylose bind site (xylO2 sequence), one half RSR fragment and the other half RSR fragment were amplified by annealed primer pair pxyla-F/pxyla-R, R1-F/R1-26bp-R, and R2-26bp-F/R2-R, respectively. The terminator dbt fragment was amplified from pUB-sfGFP using primer pair RSR dbt-F/RSR dbt-R. The four fragments were fused with fusion PCR to generate T26 mini-CRISPR fragment. Similarly, P_{xylA} promoter containing another xylose bind site (xylO2 sequence), one half RSR fragment and the other half RSR fragment were amplified by annealed primer pair pxyla-F/pxyla-R, R1-F/R1-27bp-R, and R2-27bp-F/R2-R, respectively. The terminator dbt fragment was amplified from pUB-sfGFP using primer pair RSR dbt-F/RSR dbt-R. The four fragments were fused with fusion PCR to generate T27 mini-CRISPR fragment. Finally, T26 mini-CRISPR fragment and T27 mini-CRISPR fragment were assembly with backbone amplified from pdonor plasmid by primer pair KIdonor-F/KI donor-R to generate plasmid pUB-T26KI, and pUB-T27KI, respectively.

For identification of PAM, several plasmids were constructed. C2S19 protospacer, including sixteen 5 bp upstream sequence (i.e., NNTTA), were inserted into the pUCG3.8 backbone digested with EcoRI and HindIII to generated plasmid pAATTA, pACTTA, pAGTTA, pATTTA, pCAATTA, pCCTTA, pCGTTA, pCTTTA, pGATTA, pGCTTA, pGGTTA, pGTTTA, pTATTA, pTCTTA, pTGTTA,and pTTTTA. C2S19 protospacer, including nine 3 bp upstream sequence (i.e., ATA, CTA, ATA, TAA, TCA, TGA, TTT, TTC, TTG), were insert into the pUCG3.8 backbone digested with EcoRI and HindIII to generated plasmid pATA, pCTA, pATA, pTAA, pTCA, pTGA, pTTT, pTTC and pTTG.

For pPAMtest plasmid, firstly, we constructed a plasmid pZH04-sfGFP. A sfGFP fragment containing RBS and terminator sequence was amplified from pUB-sfGPF plasmid using primer pair RBSGFPT-F/RBSGFPT-R. P_{xylA}* fragment was amplified by annealed primer pair pxyla*-F/pxyla*-R. Then the two fragments were assembly with plasmid backbone amplified from pZH04-xyla25 using primer pair PAMtest V-F/PAMtest V-R to generate plasmid pZH04-sfGFP. Secondly, Xyla25 fragment was obtained using annealed primer pair xyla25-F/xyla25-R. The fragment was ligated into plasmid pZH04-sfGFP digested with BsaI to obtain plasmid pPAMtest.

For the editing plasmid pZH01, *repB* fragment and Amp^R fragment were amplified from pUB-sfGFP plasmid using primer pair repB-F/repB-R and amp-F/amp-R, respectively. The xylR fragment was amplified from genome of *P. thermoglucosidasius* using primer pair xylR-F/xylR-R. Then the three fragments were assembled to generate parental plasmid pxylR. Terminator fragment was amplified from pUB-sfGFP using primer pair dbt-F/dbt-R. LacZ fragment was amplified from pCas using primer pair lacZ-F/lacZ-R. P_{xylA} fragment was amplified using annealed primer pair pxyla-F/pxyla-R. The repB fragment, Amp^R fragment, lacZ fragment, P_{xyla} fragment and plasmid backbone amplified from parental plasmid pxylR using primer pair pZH01V-F/pZH01V-R were assembled to generate plasmid pZH01.

For the plasmid pCasIB, firstly, Cas8753 fragment, chl^R fragment, ori fragment, and Cas6 fragment were amplified from pNW33n-GTIB plasmid using primer pair Cas8753-F/Cas8753-R, chl-F/chl-R, ori-F/ori-R, and Cas6-F/Cas6-R, respectively. Then, three fragments, which are Cas6, chl^R, and ori fragment were fused with fushion PCR to generate long fragment. Finally, the long fragment and Cas8753 fragment were assembly to generate pCasIB plasmid.

To delete amylase gene (*BCV53_04180*) in *P. thermoglucosidasius* NCIMB 11955, firstly, the spacer fragment was inserted into the pZH01 plasmid backbone digested with Esp3I to generate plasmid pZH01-amyl guide. Then, the upstream and downstream fragments of the amylase gene were amplified from *P. thermoglucosidasius* genomic DNA using primer pair amyl UF/amyl UR and primer pair amyl DF/amyl DR, respectively. The two fragments and plasmid backbone amplified from plasmid pZH01-amyl guide using primer pair CrV-F/CrV-R were assembled to generate plasmid pZH01-amylKO.

For the deletion plasmid in *E. coli* W3110, the spacer fragment was inserted into the pZH01 plasmid backbone digested with Esp3I to generated plasmid pZH01-pyrF guide. Then, the upstream and downstream fragments of the *pyrF* gene were amplified from genomic DNA of *E. coli* W3110 using primer pair pyrF UF/ pyrF UR and pyrF DF/ pyrF DR, respectively. The two fragments and plasmid backbone amplified from plasmid pZH01-amyl guide using primer pair CrV-F/CrV-R were assembled to generate plasmid pZH01-pyrFKO.

The plasmid pZH01-5arrayKI was used for the construction of supercompetent strain. Eight fragments, including inducible promoter, five CRISPR-array and terminator, were got by annealed primer pair Com 1-F/Com 1-R, Com 2-F/Com 2-R, Com 3-F/Com 3-R, Com 4-

F/Com 4-R, Com 5-F/Com 5-R, Com 6-F/Com 6-R, Com 7-F/Com 7-R and Com 8-F/Com 8-R. Vector fragment was amplified from plasmid pUCG3.8 using primer pair pUCG3.8 V-F/pUCG3.8 V-R. Then the eight fragments phosphorylated with PNK were ligated into the vector fragment digested with BsaI using T4 ligase to obtain intermediate plasmid pUCG3.8-5guide. Then, the spacer fragment, obtained by annealed primer pair Spacer-F/Spacer-R, was inserted into the pZH01 plasmid backbone digested with Esp3I to generate plasmid pZH01-spacer. Finally, the upstream and downstream fragments of the *BCV53_9960* gene were amplified from *P. thermoglucosidasius* genomic DNA using primer pair 9960 UF/9960 UR and primer pair 9960 DF/9960 DR, respectively. The CRISPR-array fragment was amplified from intermediate plasmid pUCG3.8-5guide using primer pair 5array-F/5array-R. The upstream and downstream fragments of the *BCV53_9960* gene and the CRISPR-array fragment were fused with fusion PCR to generate donor DNA. The donor DNA was assembly with plasmid backbone amplified from plasmid pZH01-spacer using primer pair pZH01-F/pZH01-R to generate plasmid pZH01-5arrayKI.

Supplementary Note 1

Glc: glucose, G6P: glucose 6-phosphate, PYR: pyruvate, Ac-CoA: acetyl-CoA, TCA: tricarboxylic acid cycle, αKG: Oxoglutarate, Oxa: Oxaloacetate, Ru5P: ribulose-5-phosphate, PRPP: 5-phospho-α-D-ribosyl-1-pyrophosphate, PRA: 5-phospho-α-D-ribosylamin, IMP: inosine 5'-monophosphate, SAMP: succinyl adenosine monophosphate, XMP: xanthine 5'-monophosphate, GTP: guanosine 5'-triphosphate. FMN: Flavin Mononucleotide.

Supplementary Note 2

```
# this code is used to generate tgRNAs for CRISPRi library
import re
datafin = []

with open(r'F:\GCA_001700985.1_ASM170098v1_cds_from_genomic.txt')as file_object:
    data1 = file_object.read()
    data2 = data1.split('>')
    for data in data2:
        p = data.replace('\n', '')
        if len(p) > 0:
            i = p.rindex(']')
            c = p[i + 1:]
            m = len(c) - 20
            x = 'TTA'
            q = [x.start() for x in re.finditer(x, c[0:m])]
            n = len(q)
            datafin.append('>')
            datafin.append(p[:i+1])
            datafin.append('\n')
            if n > 4:
                i = q[2]
                r = c[i + 3:i + 23]
                datafin.append(r)
                datafin.append('\n')

                i = q[n - 1]
                r = c[i + 3:i + 23]
                datafin.append(r)
                datafin.append('\n')
```

```
                i = q[int((n + 1) / 2)]
                r = c[i + 3:i + 23]
                datafin.append(r)
                datafin.append('\n')

            else:
                for i in q[:3]:
                    r = c[i + 3:i + 23]
                    datafin.append(r)
                    datafin.append('\n')

f2 = ('re-TTAfinal.txt')
with open(f2, 'w')as file_object:
    file_object.writelines(datafin)
print('fine!')
```

Uncropped gels

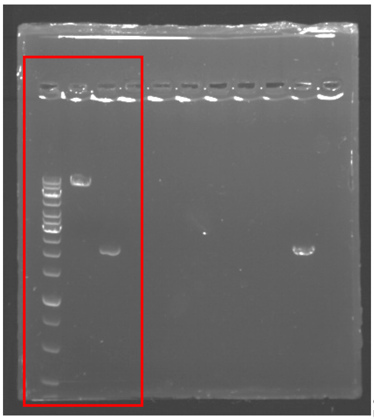
Supplementary Figure 2a

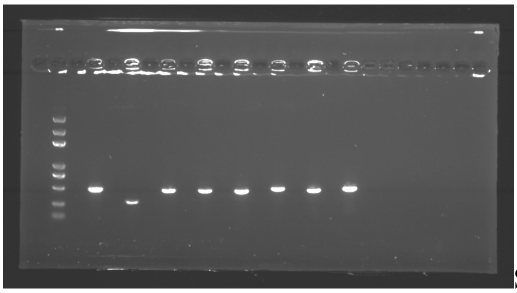
Supplementary Figure 2b

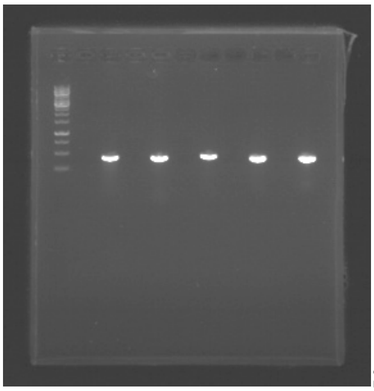
Supplementary Figure 3b

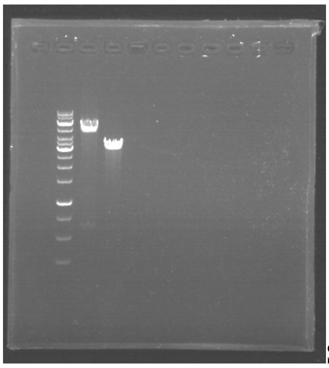
Supplementary Figure 7b

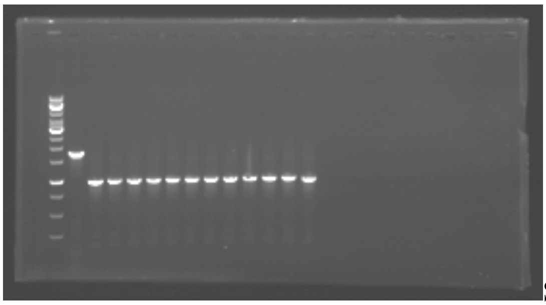
Supplementary Figure 9b

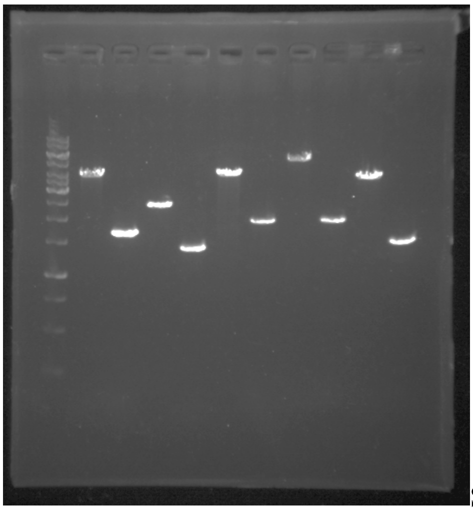
Supplementary Figure 10a

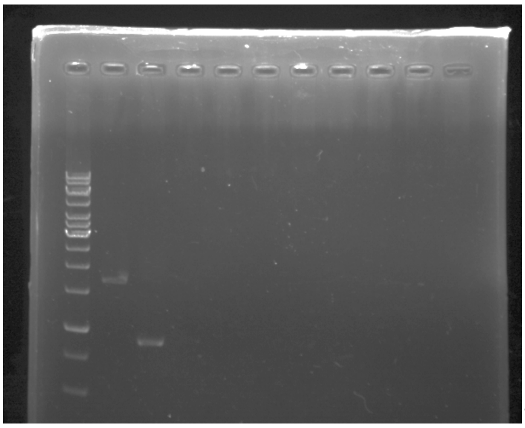
Supplementary Figure 10e

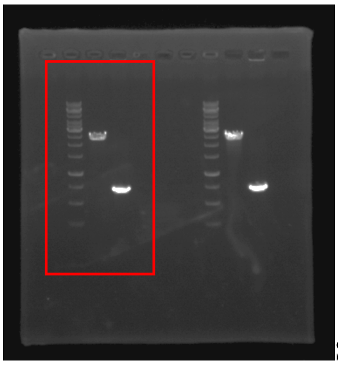
Supplementary Figure 10g

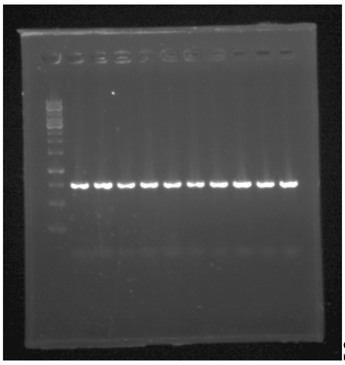
Supplementary Figure 10i

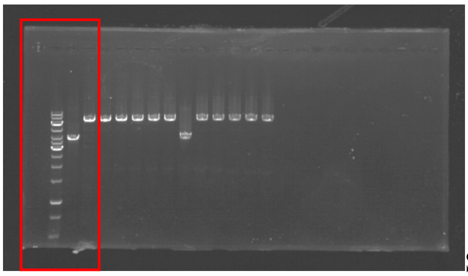
Supplementary Figure 11a

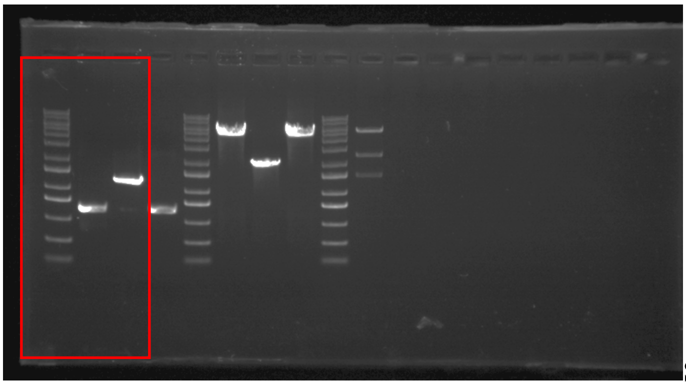
Supplementary Figure 11g

References

1. Mougiakos I, *et al.* Characterizing a thermostable Cas9 for bacterial genome editing and silencing. *Nature communications* **8**, 1647 (2017).
