# Supplementary material for: A thermostable type I-B CRISPR-Cas system for orthogonal and multiplexed genetic engineering
Source: Nat Commun. 2023 Oct 4;14:6193. doi: 10.1038/s41467-023-41973-5 (PMC10551041; doi:10.1038/s41467-023-41973-5)
Supplement: Supplementary file 2 — Reporting Summary [file 41467_2023_41973_MOESM2_ESM.pdf]

nature portfolio

Corresponding author(s): Lixin Zhang, Weishan Wang

Last updated by author(s): sep. 21, 2023

Reporting Summary

Nature Portfolio wishes to improve the reproducibility of the work that we publish. This form provides structure for consistency and transparency in reporting. For further information on Nature Portfolio policies, see our Editorial Policies and the Editorial Policy Checklist.

Statistics

For all statistical analyses, confirm that the following items are present in the figure legend, table legend, main text, or Methods section.

n/a	Confirmed	
☐	☒	The exact sample size (*n*) for each experimental group/condition, given as a discrete number and unit of measurement
☐	☒	A statement on whether measurements were taken from distinct samples or whether the same sample was measured repeatedly
☐	☒	The statistical test(s) used AND whether they are one- or two-sided<br>*Only common tests should be described solely by name; describe more complex techniques in the Methods section.*
☒	☐	A description of all covariates tested
☒	☐	A description of any assumptions or corrections, such as tests of normality and adjustment for multiple comparisons
☐	☒	A full description of the statistical parameters including central tendency (e.g. means) or other basic estimates (e.g. regression coefficient) AND variation (e.g. standard deviation) or associated estimates of uncertainty (e.g. confidence intervals)
☐	☒	For null hypothesis testing, the test statistic (e.g. F, t, r) with confidence intervals, effect sizes, degrees of freedom and P value noted<br>*Give P values as exact values whenever suitable.*
☒	☐	For Bayesian analysis, information on the choice of priors and Markov chain Monte Carlo settings
☒	☐	For hierarchical and complex designs, identification of the appropriate level for tests and full reporting of outcomes
☒	☐	Estimates of effect sizes (e.g. Cohen's *d*, Pearson's *r*), indicating how they were calculated

Our web collection on statistics for biologists contains articles on many of the points above.

Software and code

Policy information about availability of computer code

Data collection	python (version: 3.10.4) was used for design of genome-wide tgRNA library.
Data analysis	GraphPad Prism (version 8.4.0), Microsoft Excel (version 16.45), SnapGene (version 6.0.2)

For manuscripts utilizing custom algorithms or software that are central to the research but not yet described in published literature, software must be made available to editors and reviewers. We strongly encourage code deposition in a community repository (e.g. GitHub). See the Nature Portfolio guidelines for submitting code & software for further information.

Data

Policy information about availability of data

All manuscripts must include a data availability statement. This statement should provide the following information, where applicable:

- Accession codes, unique identifiers, or web links for publicly available datasets
- A description of any restrictions on data availability
- For clinical datasets or third party data, please ensure that the statement adheres to our policy

The RNA-seq data are available in the NCBI Short Read Archive with BioProject ID PRJNA1018439. The integrated software used for tgRNA library design can be found at https://zenodo.org/account/settings/github/repository/Wang-CASlab/tgRNA-library.

Research involving human participants, their data, or biological material

Policy information about studies with human participants or human data. See also policy information about sex, gender (identity/presentation), and sexual orientation and race, ethnicity and racism.

Reporting on sex and gender	N/A
Reporting on race, ethnicity, or other socially relevant groupings	N/A
Population characteristics	N/A
Recruitment	N/A
Ethics oversight	N/A

Note that full information on the approval of the study protocol must also be provided in the manuscript.

Field-specific reporting

Please select the one below that is the best fit for your research. If you are not sure, read the appropriate sections before making your selection.

[x] Life sciences [ ] Behavioural & social sciences [ ] Ecological, evolutionary & environmental sciences

For a reference copy of the document with all sections, see nature.com/documents/nr-reporting-summary-flat.pdf

Life sciences study design

All studies must disclose on these points even when the disclosure is negative.

Sample size	To assess transformation efficiency, each experimental condition was represented by three biological replicates. This choice of three replicates was based on standard practices in the field, which have shown that this number provides a reasonable balance between precision and resource constraints.<br>For the measurement of transcriptional repression using fluorescence intensity, we employed a more detailed approach. Each experimental condition included three biological replicates, and to enhance precision, each biological replicate was further divided into two technical replicates. This decision was made to account for potential variability in technical measurements, ensuring the accuracy and reproducibility of our fluorescence intensity data.<br>When assessing the growth rate of P. thermoglucosidasius, we performed three biological replicates. This sample size was chosen based on the practical considerations of working with this particular strain and ensuring the robustness of our growth rate measurements.<br>It is important to note that our experimental design was flexible in response to the magnitude of error observed during the initial experiments. When substantial variability or uncertainty was encountered, we expanded the number of independent replicates to ensure the reproducibility of our findings.
Data exclusions	No data was excluded
Replication	All experiments were repeated more than three times and the presented data are reproducible.
Randomization	Genes edited in the strain were selected for assessment of editing efficiency using colony PCR or Sanger sequencing through random selection.
Blinding	This study does not involve procedures that require blinding, as all data were collected to corresponding to the designated plasmids, phenotype, and genotype of the P. thermoglucosidasius or E. coli clones.

Reporting for specific materials, systems and methods

We require information from authors about some types of materials, experimental systems and methods used in many studies. Here, indicate whether each material, system or method listed is relevant to your study. If you are not sure if a list item applies to your research, read the appropriate section before selecting a response.

Materials & experimental systems

n/a	Involved in the study
☒	☐ Antibodies
☒	☐ Eukaryotic cell lines
☒	☐ Palaeontology and archaeology
☒	☐ Animals and other organisms
☒	☐ Clinical data
☒	☐ Dual use research of concern
☒	☐ Plants

Methods

n/a	Involved in the study
☒	☐ ChIP-seq
☒	☐ Flow cytometry
☒	☐ MRI-based neuroimaging
