# Supplementary material for: A thermostable type I-B CRISPR-Cas system for orthogonal and multiplexed genetic engineering
Source: Nat Commun. 2023 Oct 4;14:6193. doi: 10.1038/s41467-023-41973-5 (PMC10551041; doi:10.1038/s41467-023-41973-5)
Supplement: Supplementary file 3 — Description of Additional Supplementary Files [file 41467_2023_41973_MOESM3_ESM.pdf]

Description of Additional Supplementary Files

File Name: Supplementary Data 1

Description: Spacers in the seven CRISPR arrays

File Name: Supplementary Data 2

Description: a genome-scale CRISPR interference library with 11281 truncated guide RNAs (tgRNAs)

File Name: Supplementary Data 3

Description: Strains used and constructed in this study

File Name: Supplementary Data 4

Description: Primers used in this study

File Name: Supplementary Data 5

Description: Abundance changes of tgRNAs assayed by deep sequencing
